# Association between personal values in adolescence and mental health and well-being in adulthood: a cross-cultural study of working populations in Japan and the United States

**DOI:** 10.1186/s12991-020-0260-4

**Published:** 2020-02-11

**Authors:** Kazuhiro Watanabe, Norito Kawakami, Daisuke Nishi

**Affiliations:** Department of Mental Health, Graduate School of Medicine, The University of Tokyo, 7-3-1 Hongo, Bunkyo-ku, Tokyo, 113-0033 Japan

**Keywords:** Well-being, Values, Adolescence, Youth, Workers, Health-related quality of life

## Abstract

**Background:**

For promoting mental health and well-being of individuals, it is important to investigate its association with personal values. However, in Eastern Asian countries, no study has yet investigated the association between personal values in adolescence and mental health and well-being in adulthood. To fill that research gap, we conducted a cross-sectional study based on two online surveys of working populations in Japan and the United States.

**Methods:**

A total of 516 workers from each of the two countries, aged 30–49 years, completed a questionnaire that measured personal values in adolescence, current psychological distress, health-related quality of life, and subjective well-being (satisfaction and happiness). Personal values were measured by items based on Schwartz’s theory of basic values and people’s commitment to those ten values. Multiple group path analysis was performed to examine the associations between personal values in adolescence and health-related outcomes, grouped by country.

**Results:**

Care, graduating from school, and commitment to values were associated with better mental health and well-being in Japanese participants. Belief and challenging were associated with better mental health and well-being in US participants. On the other hand, financial success was associated with poor mental health and well-being in Japanese participants. Avoiding causing trouble and positive evaluation were associated with poor mental health and well-being in the US participants.

**Conclusions:**

Certain personal values and commitment to those values in adolescence may be associated with mental health and well-being in adulthood. To address the limitations of this study, future studies should use a longitudinal design and investigate the interactions among the types of personal values and commitment to the values.

## Background

There is a growing worldwide interest in the promotion of mental health and well-being. Adverse mental health conditions, such as depressive disorder, make a substantial independent contribution to the global disease burden [1]. Well-being is distinguished by the absence of negative factors, such as depression, anxiety, distress, and negative affect, and is independently correlated with low mortality risk [2–4]. Recently, the United Nations (UN) included mental health and well-being as one of its 17 Sustainable Development Goals (SDGs) [5]. Therefore, we cannot consider health without considering the promotion of mental health and well-being.

The association between mental health and well-being and personal values has repeatedly been investigated in Western countries, Australia, and the Middle East [6–20]. Personal values are defined as broad, desirable, and trans-situational goals that underlie and guide attitudes and behavior [18, 21]. Schwartz [22] proposed that the content and structure of personal values consist of ten motivationally distinct priorities: power, achievement, hedonism, stimulation, self-direction, universalism, benevolence, tradition, conformity, and security. Based on Schwartz’s theory, the associations between personal values and health have been discussed from three perspectives: healthy values, goal attainment, and value congruence [6]. For example, Sagiv and Schwartz [16] reported that in Israeli and German people, stimulation, self-direction, and achievement are positively related to subjective well-being (positive affect), while security, conformity, and tradition are negatively related to the subjective well-being. Bobowik et al. [6] also investigated the association between personal values and subjective well-being among Europeans and found that the values hedonism, stimulation, and self-direction were positively associated, while power, tradition, and conformity were negatively associated, with subjective well-being. On the other hand, a study of Native American youth [14] indicated that tradition-based or benevolent values were negatively associated with depressive symptoms. Cultural contexts seem to moderate individual-level associations between personal values and subjective well-being [17, 18]. In summary, values related to openness to change (self-direction, stimulation, and hedonism), achievement, and benevolence tend to be positively associated, while those related to conservatism (tradition, conformity, and security) and power tend to be negatively associated with mental health and well-being. In addition, these associations may be moderated by higher-level factors, such as a country’s level of development, egalitarianism, tight cultures [17], and individualism [23]. Furthermore, not only *what* goals people pursue (i.e., what types of values they prioritize), but also *why* people pursue them (i.e., whether they prioritize autonomous or controlled motives) may independently correlate with mental health and well-being [24]. Therefore, *how* people commit themselves to values they deem important could be another aspect of personal values.

However, such associations have yet to be investigated in East Asian countries. Further, all previous studies have examined associations cross-sectionally at a given time point, while no studies have investigated how personal values in adolescence are correlated with mental health and well-being in adulthood. Adolescence is a period of substantial psychological and emotional development during which the brain’s reward system is remodeled [25], and studies on personal values in adolescence can provide important insights into adult mental health and well-being. The study could also be useful for investments in adolescence, which can have a number of benefits in adult life [25].

This study was conducted among healthy adult working populations of Japan and the United States. We investigated the associations between personal values in adolescence and psychological distress, health-related quality of life (HRQoL), and subjective well-being (life satisfaction and happiness). This study is the first in Eastern Asian countries to investigate the associations between personal values in adolescence and mental health and well-being in adulthood. We hypothesized that for participants from a Western culture (the US), personal values related to openness to change, achievement, and benevolence would be positively associated with mental health and well-being, while those related to conservatism and power would be negatively associated. On the other hand, this would differ among participants from an Eastern culture (Japan). How people commit themselves to values they consider important would also be positively associated with mental health and well-being.

## Methods

### Study design and setting

This study was a cross-sectional study based on two online surveys conducted in Japan (February 2017) and the United States (July 2017). We recruited workers aged 30–49 years through an Internet survey company [26]. The company has access to more than 2,000,000 registered members who live in all prefectures in Japan and various states in the US. Eligible workers (described below) were randomly recruited from the member pool, stratified by gender (men, women), age range (30–34, 35–39, 40–44, 45–49), and area (in the US, the East and West Coasts). The East Coast included the states of Delaware, Maryland, New York, New Jersey, and Pennsylvania, and the West Coast included the states of California, Oregon, and Washington. Participants were asked to complete a questionnaire that measured personal values in adolescence, psychological distress, HRQoL, and subjective well-being in adulthood. Participants evaluated personal values by recalling their adolescent period. We obtained online informed consent from all participants by asking the participants to read terms and conditions and to press “agree”. The survey did not ask for personal data (e.g., names, e-mail addresses) from the participants. The study protocol was approved by the research ethics committee of the Graduate School of Medicine and the Faculty of Medicine, The University of Tokyo, Japan [No. 2953-(3) for the survey in Japan and 10003-(5) for that in the US]. This article complies with the Strengthening the Reporting of Observational studies in Epidemiology (STROBE) guidelines [27].

### Participants

A total of 1032 individuals, 516 each from Japan and the US, were recruited from those who registered with the Internet survey company. Participant inclusion criteria were (a) workers who lived in Japan or the US who (b) were between 30 and 49 years old. There were no exclusion criteria; we included all workers regardless of their employment status, shift type, occupation, or industry. Based on these criteria, the Internet survey company randomly recruited workers from their potential pool of participants via e-mail and their own website. If the eligible workers agreed to the terms and conditions of the online survey, they could access and complete the self-report questionnaire on a first-come, first-served basis. Because the Internet survey company stopped recruitment once the target number of respondents had been reached, the response rate could not be determined. Participants were awarded approximately 50 “Macromill points” as a reward for participation in each survey, which could be cashed in and used to shop online (one point was equivalent to one Japanese yen).

### Measurements

All variables in this study (personal values in adolescence, psychological distress, HRQoL, subjective well-being, and demographic variables) were measured by an online self-report questionnaire.

#### Personal values in adolescence

Participants recalled their personal values from adolescence, which we measured by value priorities and degree of commitment to those values. For the measurement of value priorities, we used 11 items [28] from the 57-item Portrait Values Questionnaire (PVQ-57) [29], which has good cross-cultural validity including Japan. Although widely used to assess value priorities, the scale has generally been used for adults. The terms and constructs for measuring value priorities in adolescence should be locally meaningful [14]; moreover, these might differ from those for adults. For measuring value priorities in children, the Picture-Based Value Survey for Children (PBVS-C) [30] has already been developed. However, it is difficult to compare the scores of value priorities directly between children and adults based on the picture-based scale. Therefore, we selected and revised PVQ-57 items to be suitable to assess value priorities in adolescence. First, based on Schwartz’s 10 motivationally distinct priorities, we selected and revised eight items from the PVQ-57: avoiding causing trouble, positive evaluation, beliefs, improving society, social influence, challenging, care, and stable lifestyle. “Tradition” was not included because it could be correlated to religiosity [31] and it would be difficult to compare religiosity between Japanese and American people [32, 33]. Second, we developed three additional items that could motivate behavior, especially in adolescence: financial success, interest, and graduating from school. These 11 items, chosen to measure value orientations, were rated on a seven-point Likert scale (1 = not at all, 7 = very important) based on the question, “When you were 15 to 16 years old, how important did you think the following *values* were in your life?”

Commitment to values was measured by the Personal Values Questionnaire II (PVQ-II) [33, 34]. This scale is used in clinical research of acceptance and commitment therapy and measures commitment to and motivation regarding the values that people consider important. The original and Japanese PVQ-II consists of 9 and 8 items, respectively (e.g., “How committed are you to living this value?”); the items are rated on a five-point Likert scale. The internal consistency, concurrent, and structural validity has already been confirmed both for the English and Japanese versions of the PVQ-II [34, 35]. In this study, we revised the items to the past tense and instructed the participants to answer the items they considered the most important when they were 15 to 16 years old. The total scores on the PVQ-II were used for analysis; higher scores indicated more commitment to important values. Because there was one less item in the Japanese PVQ-II than in the English PVQ-II, the scores of Japanese participants were multiplied by 9/8.

#### Psychological distress

General psychological distress was measured using the K6 scale [36, 37]. The scale consists of six items (e.g., “About how often did you feel nervous?”) about how often participants had experienced symptoms of psychological distress during the last 30 days. All items were rated on a five-point Likert scale (0 = none of the time, 4 = all the time). The reliability and validity of both the English and Japanese versions of the K6 were confirmed in a previous study [36, 37]. In this study, total continuous scores were used for analyses; higher scores indicated more severe psychological distress.

#### HRQoL

HRQoL was measured using the eight-item Short-Form Health Survey (SF-8) [38, 39]. This commonly used scale [40] consists of eight items that measure eight domains of HRQoL: physical functioning, physical role, bodily pain, general health, vitality, social functioning, emotional role, and mental health. In this study, nationally standardized scores for physical and mental domains of HRQoL were calculated using a norm-based scoring system in Japan and the US. By using this system, the mean score and standard deviation were transformed to 50 and 10, respectively, based on the national standard values. Higher scores indicated a higher HRQoL.

#### Subjective well-being

Subjective well-being was measured by two items: life satisfaction and happiness. Participants were asked, “How satisfied are you with your current life?” The item was rated on a four-point Likert scale (1 = dissatisfied, 4 = satisfied), and a higher score indicated higher satisfaction. A question taken from the PERMA-Profiler [41] was asked, “Taking all things together, how happy would you say you are?” The PERMA-Profiler is the measure of well-being based on Seligman’s theory [42] and its reliability and validity are confirmed both in English and Japanese versions [43]. The item was rated on an 11-point Likert scale (0 = not at all, 10 = completely), and a higher score indicated higher happiness.

### Sample size calculation

The required sample size was calculated taking into account the possible correlations (*ρ*) between personal values in adolescence and outcomes in adulthood (i.e., psychological distress, health-related quality of life, and subjective well-being). Based on previous studies [6–20], the estimated minimum correlation was set to 0.1. Using G*Power version 3.1.9.2 [44], the required sample size was estimated at 1043, for an α error probability of 0.05 and a power (1 − *β*) of 0.90. If we collect data from 1032 participants and analyze the data, post hoc statistical power of analysis (1 − *β*) would be 0.897. If we analyze the data stratified by countries (i.e., 516 in Japan and 516 in the US), post hoc statistical power of analysis (1 − *β*) would be 0.625.

### Statistical analysis

**Fig. 1:**
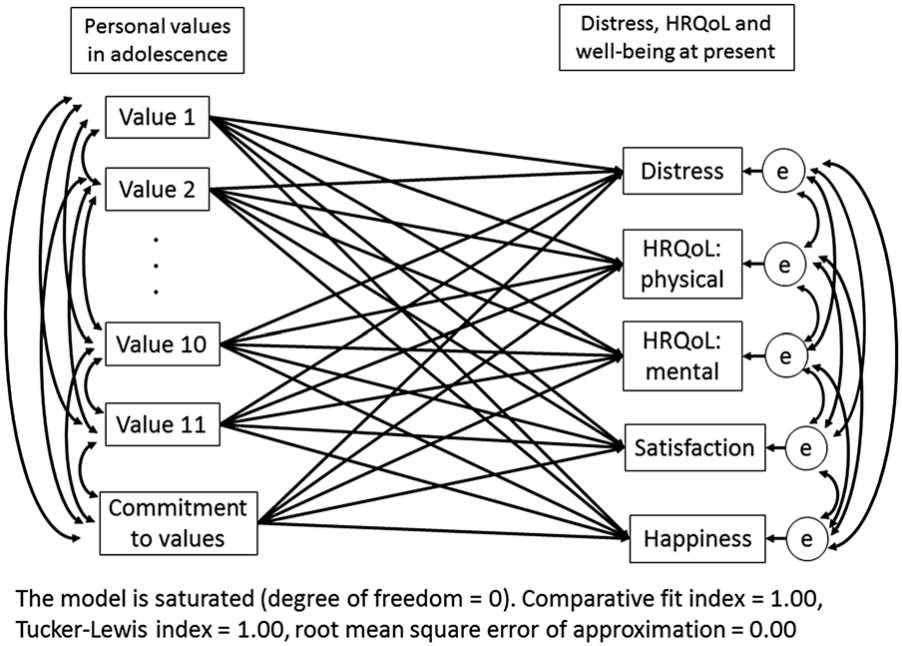
A tested model for a multiple group path analysis

Descriptive statistics for the participants stratified by country and bivariate correlation coefficients (*r*) between personal values and the outcomes were calculated. Since we calculated correlation coefficients 136 times in each country, the significance level was reduced to 1/136 (i.e., 0.037%) using Bonferroni’s method. For the main analysis, multiple group path analysis was performed, grouped by the countries (Japan and the US). We tested a direct association model between personal values in adolescence and each outcome in adulthood (Fig. 1). Covariates were assumed among all variables of personal values and among the outcomes. There was no equality constraint on the parameters between the models in Japan and the US, assuming that each association should be estimated differently. Because the model was saturated, its model fit indices were not considered (CFI = 1.00, TLI = 1.00, RMSEA = 0.00). PASW statistics version 18 (IBM SPSS software) and Mplus version 7.4 [45] were used for the analyses.

## Results

### Participants’ characteristics

Table 1 shows the characteristics of the Japanese participants (n = 516) and the US participants (*n* = 516). There were no missing responses to the items because the survey required the participants to answer all items of the questionnaire. In both countries, most participants were full-time (56.8% in Japan and 73.3% in the US), daytime (87.2% in Japan and 79.8% in the US) workers. Approximately 70% of the US participants were European-American, with a smaller secondary majority of Hispanic/Latinx Americans (9.1%). There were significant demographic differences between the US and Japan. In terms of educational status, the number of participants who graduated from a university and/or graduate school was significantly larger in the US than in Japan. The number of regular full-time workers was significantly higher in the US than in Japan, while part-time workers were significantly more common in Japan. The number of workers with a day-time shift was significantly higher in Japan than in the US, while those with rotation- and night-shifts were more common in the US. Participants in clerical and productive jobs were significantly more common in Japan than in the US, while participants in managerial and professional/technical jobs were more common in the US.

**Table 1:** Characteristics of the participants

	Japan (*N* = 516)	US (*N* = 516)	*p* for difference
	*N* (%)	*N* (%)	
Gender (male)	260 (50.4)	258 (50.0)	–
Age range (years)			–
30–34	129 (25.0)	132 (25.6)	
35–39	129 (25.0)	128 (24.8)	
40–44	129 (25.0)	128 (24.8)	
45–49	129 (25.0)	128 (24.8)	
Ethnicity (US only)			–
European Americans (white)	–	358 (69.4)	
African Americans	–	24 (4.7)	
Hispanic and Latinx Americans	–	47 (9.1)	
Asian Americans	–	38 (7.4)	
Native Americans	–	30 (5.8)	
Others	–	19 (3.7)	
Educational status			
Junior high/middle/high school	157 (30.4)	46 (8.9)	< 0.001
College/vocational school	127 (24.6)	72 (14.0)	
University/graduate school	221 (42.8)	388 (75.2)	
Others/refusal to respond	11 (2.1)	10 (2.0)	
Employment status			
Regular/full-time	293 (56.8)	378 (73.3)	< 0.001
Part-time	133 (25.7)	71 (13.8)	
Contract/dispatched	50 (9.7)	39 (7.6)	
Freelancer	34 (6.6)	12 (2.3)	
Others	6 (1.2)	16 (3.1)	
Shift type			0.004
Day-time shift	450 (87.2)	412 (79.8)	
Rotation-shift	55 (10.6)	81 (15.7)	
Night-shift	11 (2.1)	23 (4.5)	
Occupation			< 0.001
Managerial	41 (7.9)	142 (27.5)	
Professional/technical	105 (20.3)	221 (42.8)	
Clerical	162 (31.4)	45 (8.7)	
Sales/services	73 (14.1)	19 (3.7)	
Transport	14 (2.7)	7 (1.4)	
Production	77 (14.9)	12 (2.3)	
Agriculture/forestry/fisheries	5 (1.0)	0 (0.0)	
Others	39 (7.6)	35 (6.8)	

### Bivariate correlations between personal values and outcomes

Table 2 shows the Pearson’s correlation coefficients (*r*) for each component of personal values in adolescence, current psychological distress, HRQoL, and subjective well-being. Among the components of personal values, value orientations and commitment to values showed consistent positive associations with each other for both countries. The associations between outcomes and personal values were different between the Japanese and the US participants. For Japanese participants, psychological distress had no significant correlation with personal values. However, for the US participants, many of the value orientations were significantly and positively associated with psychological distress. None of the personal values had significant positive associations with HRQoL among Japanese participants. In the US, however, value orientations were negatively associated with physical and mental components of HRQoL. For Japanese participants, subjective well-being had significant positive associations with care and commitment to values. In the US, many of the other value orientations had significant and positive associations with subjective well-being.

**Table 2:** Bivariate correlations between personal values in adolescence and the outcomes in adulthood in Japan and the US (*N* = 516 in each country)

Japan	Mean (SD)	1	2	3	4	5	6	7	8	9	10	11	12	13	14	15	16	17
US																		
1. Avoiding causing trouble	5.04 (1.5)	1.00																
	4.86 (1.8)	1.00																
2. Positive evaluation	4.56 (1.5)	0.42*	1.00															
	4.97 (1.7)	0.49*	1.00															
3. Belief	4.51 (1.5)	0.39*	0.46*	1.00														
	5.00 (1.7)	0.43*	0.48*	1.00														
4. Financial success	3.97 (1.6)	0.25*	0.27*	0.47*	1.00													
	5.08 (1.7)	0.38*	0.36*	0.59*	1.00													
5. Improving society	3.35 (1.5)	0.33*	0.32*	0.47*	0.53*	1.00												
	4.81 (1.8)	0.43*	0.35*	0.65*	0.72*	1.00												
6. Interest	4.95 (1.4)	0.25*	0.36*	0.53*	0.34*	0.30*	1.00											
	5.57 (1.4)	0.35*	0.34*	0.54*	0.49*	0.55*	1.00											
7. Social influence	3.35 (1.5)	0.23*	0.38*	0.46*	0.52*	0.68*	0.35*	1.00										
	4.83 (1.7)	0.38*	0.38*	0.63*	0.63*	0.79*	0.55*	1.00										
8. Challenging	4.32 (1.6)	0.27*	0.41*	0.62*	0.37*	0.46*	0.58*	0.52*	1.00									
	5.02 (1.5)	0.36*	0.32*	0.58*	0.53*	0.64*	0.56*	0.74*	1.00									
9. Care	4.95 (1.5)	0.53*	0.32*	0.48*	0.28*	0.32*	0.40*	0.25*	0.47*	1.00								
	5.32 (1.5)	0.45*	0.35*	0.59*	0.48*	0.53*	0.47*	0.54*	0.63*	1.00								
10. Graduating from school	4.11 (1.6)	0.30*	0.45*	0.34*	0.37*	0.38*	0.27*	0.36*	0.40*	0.30	1.00							
	4.63 (1.9)	0.28*	0.35*	0.44*	0.50*	0.54*	0.36*	0.56*	0.49*	0.43	1.00							
11. Stable lifestyle	4.53 (1.5)	0.41*	0.33*	0.38*	0.60*	0.41*	0.35*	0.38*	0.33*	0.46	0.53*	1.00						
	5.38 (1.5)	0.47*	0.32*	0.53*	0.62*	0.60*	0.53*	0.62*	0.58*	0.66	0.46*	1.00						
12. Commitment to values	27.94 (5.2)	0.05	0.08	0.36*	0.13	0.06	0.48*	0.06	0.40*	0.35	0.08	0.15	1.00					
	31.47 (4.4)	0.18*	0.23*	0.45*	0.42*	0.47*	0.42*	0.51*	0.49*	0.41	0.35*	0.45*	1.00					
13. Psychological distress	5.58 (5.4)	− 0.04	− 0.00	− 0.03	0.07	− 0.04	− 0.04	− 0.01	− 0.04	− 0.09	− 0.09	0.02	− 0.10	1.00				
	8.16 (6.5)	0.22*	0.21*	0.10	0.13	0.18*	0.05	0.19*	0.12	0.04	0.20*	0.10	0.03	1.00				
14. HRQoL: physical	48.44 (6.3)	0.03	0.09	− 0.01	− 0.04	− 0.05	− 0.04	− 0.03	0.00	0.04	0.10	0.03	0.04	− 0.22*	1.00			
	47.38 (8.8)	− 0.16*	− 0.17*	− 0.08	− 0.05	− 0.11	− 0.01	− 0.13	− 0.09	− 0.06	− 0.11	− 0.02	0.02	− 0.55*	1.00			
15. HRQoL: mental	45.85 (8.0)	− 0.04	− 0.02	0.01	− 0.07	0.06	0.04	0.04	0.03	0.09	0.07	− 0.04	0.11	− 0.69*	0.01	1.00		
	45.87 (10.7)	− 0.18*	− 0.19*	− 0.06	− 0.09	− 0.08	0.02	− 0.08	− 0.06	− 0.02	− 0.12	− 0.04	0.03	− 0.72*	0.45*	1.00		
16. Satisfaction	2.43 (0.8)	0.07	0.06	0.04	− 0.05	0.10	0.01	0.07	0.09	0.15	0.10	0.01	0.12	− 0.43*	0.22*	0.43*	1.00	
	3.21 (0.8)	0.12	0.08	0.28*	0.24*	0.30*	0.15	0.32*	0.32*	0.22*	0.28*	0.25*	0.35*	− 0.18*	0.14	0.33*	1.00	
17. Happiness	5.71 (2.4)	0.10	0.08	0.07	− 0.05	0.03	0.04	0.01	0.10	0.17*	0.11	0.02	0.16*	− 0.50*	0.28*	0.42*	0.72*	1.00
	7.21 (2.1)	0.14	0.10	0.34*	0.28*	0.38*	0.21*	0.39*	0.38*	0.27*	0.36*	0.26*	0.43*	− 0.16*	0.12	0.33*	0.79*	1.00

*HRQoL* health-related quality of life

*p < 0.0037 (the significance level was reduced to 1/136 using Bonferroni’s method)

### Multiple group path analysis in Japan and the US

Table 3 shows the coefficients of the results of the multiple group path analysis (Fig. 1), which investigated multiple associations between personal values in adolescence and psychological distress, HRQoL, and subjective well-being in adulthood.

**Table 3:** Associations between personal values in adolescence and outcomes in adulthood in multiple group path analysis (*N* = 1032)

	Psychological distress				HRQoL: physical				HRQoL: mental			
	Japan		US		Japan		US		Japan		US	
	Coeff	*p*	Coeff	*p*	Coeff	*p*	Coeff	*p*	Coeff	*p*	Coeff	*p*
Avoiding causing trouble	− 0.01	0.844	*0.18*	*0.001*	0.00	0.968	*− 0.12*	*0.024*	0.02	0.767	*− 0.15*	*0.007*
Positive evaluation	0.06	0.271	*0.14*	*0.009*	0.10	0.059	*− 0.13*	*0.018*	− 0.07	0.188	*− 0.16*	*0.003*
Belief	− 0.01	0.912	− 0.07	0.287	− 0.03	0.668	0.03	0.615	− 0.04	0.556	0.04	0.544
Financial success	*0.15*	*0.018*	− 0.03	0.687	− 0.04	0.521	0.05	0.446	*− 0.14*	*0.024*	− 0.05	0.469
Improving society	− 0.08	0.236	0.08	0.302	− 0.08	0.215	− 0.05	0.514	0.10	0.109	− 0.02	0.792
Interest	− 0.02	0.779	− 0.10	0.083	− 0.10	0.080	0.10	0.086	0.03	0.609	*0.13*	*0.019*
Social influence	− 0.03	0.653	0.14	0.093	0.01	0.914	− 0.16	0.060	0.08	0.238	− 0.01	0.953
Challenging	0.07	0.265	0.03	0.674	− 0.02	0.792	− 0.03	0.722	− 0.09	0.173	− 0.07	0.348
Care	− 0.09	0.122	*− 0.16*	*0.011*	0.04	0.512	− 0.01	0.848	0.10	0.080	0.10	0.139
Graduating from school	*− 0.16*	*0.004*	*0.14*	*0.009*	*0.12*	*0.027*	− 0.06	0.301	*0.14*	*0.011*	− 0.09	0.095
Stable lifestyle	0.08	0.200	− 0.01	0.923	0.00	0.992	0.12	0.078	− 0.12	0.061	0.02	0.771
Commitment to values	− 0.10	0.060	− 0.06	0.266	0.08	0.121	0.08	0.130	*0.13*	*0.018*	0.06	0.242

	Satisfaction				Happiness			
	Japan		US		Japan		US	
	Coeff	*p*	Coeff	*p*	Coeff	*p*	Coeff	*p*
Avoiding causing trouble	0.00	0.967	0.02	0.641	0.05	0.405	0.02	0.647
Positive evaluation	0.02	0.783	*− 0.10*	*0.035*	0.03	0.625	*− 0.12*	*0.010*
Belief	− 0.08	0.224	*0.12*	*0.045*	− 0.03	0.647	*0.12*	*0.037*
Financial success	*− 0.15*	*0.014*	− 0.03	0.695	− 0.10	0.090	− 0.06	0.350
Improving society	0.12	0.058	0.06	0.470	0.02	0.779	0.12	0.093
Interest	− 0.10	0.097	*− 0.13*	*0.011*	− 0.08	0.164	*− 0.10*	*0.039*
Social influence	0.08	0.244	0.05	0.532	0.00	0.992	0.05	0.479
Challenging	0.00	0.983	*0.13*	*0.043*	0.02	0.755	*0.13*	*0.045*
Care	*0.15*	*0.010*	− 0.05	0.386	*0.14*	*0.021*	− 0.02	0.749
Graduating from school	*0.11*	*0.047*	*0.12*	*0.017*	*0.12*	*0.033*	*0.19*	*< 0.001*
Stable lifestyle	− 0.07	0.265	0.02	0.711	− 0.06	0.308	− 0.07	0.243
Commitment to values	*0.14*	*0.007*	*0.23*	*< 0.001*	*0.16*	*0.003*	*0.29*	*< 0.001*

All coefficients were standardized

*p* < 0.05 are in italic

*HRQoL* health-related quality of life, *Coeff* coefficient

In Japan, two of the value orientations showed consistent significant protective and positive associations with the following health-related outcomes: care and graduating from school. Commitment to values also had significant positive associations with subjective well-being. On the other hand, financial success had significant positive associations with psychological distress, and negative associations with HRQoL and satisfaction. The absolute values of standardized coefficients of these significant associations ranged from 0.11 to 0.16.

In the US, the results were very different from those in Japan. Two of the value orientations showed consistent, significant negative associations with the following health-related outcomes: avoiding causing trouble and positive evaluation. The other two value orientations, belief and challenging, and commitment to values were significantly and positively associated with subjective well-being. Interest and graduating from school were not consistently associated with health-related outcomes. Interest was positively associated with HRQoL and negatively associated with subjective well-being. Graduating from school was positively associated with psychological distress and subjective well-being. The absolute values of standardized coefficients of these significant associations ranged from 0.10 to 0.29.

## Discussion

The results indicated that some components of personal values in adolescence were significantly associated with current psychological distress, HRQoL, and subjective well-being. In addition, these associations were significantly different between the Japanese and the US participants. This study is the first study in Japan that examined the association between specific personal values in adolescence and mental health and well-being in adulthood.

“Care” was negatively associated with psychological distress in the US and positively associated with subjective well-being in Japan. The results suggest that this value was favorable for better mental health and well-being in both countries. As this value is related to benevolence, the results are consistent with previous studies [8–10, 12, 14]. The values “avoiding causing trouble” and “positive evaluation” were negatively associated with mental health and well-being in the US; this finding is also consistent with the findings of previous studies as well as the hypotheses of the present study. These values are related to conformity and extrinsic motivations [6, 16–18]. A new finding is that these values were not significantly associated with mental health and well-being in Japan. Possible reasons for this finding could be that the Japanese have a relatively collectivistic and tight culture (i.e., stringent norms and a low tolerance of deviant behavior) [46], and relationships with others are considered more important when adapting to the community. “Belief” and “challenging” were positively associated with subjective well-being only in the US. The results are also consistent with those of previous studies and our hypotheses because these values are related to openness to change [6, 8, 9, 12, 16–19], which is preferred in individualistic countries [6]. Therefore, prioritizing those values may lead Japanese people to feel less attuned to their society.

Contrary to our hypotheses, “financial success”, which is related to power and extrinsic motivations, was adversely associated with mental health and well-being only in Japan. In previous studies, aspiration for monetary success was negatively associated with psychological adjustment in Western countries [47]. However, the association in this study might be more complex. If a priority on financial success in adolescence is realized in adulthood, the state of mental health and well-being might improve. On the other hand, if it is not realized, that perceived failure could be detrimental for people’s self-actualization and well-being.

It is interesting that “graduating from school” was significantly associated with better mental health and well-being in both countries, while it was also positively associated with psychological distress in the US participants. The finding is inconsistent with that of a previous study and the hypotheses of the present study because this value is related to self-enhancement (power and achievement) and extrinsic motivations. The association between this value and mental health and well-being might be mediated by socioeconomic status (SES) in adulthood. Another explanation is that this value might be prevalent in both Japan and the US; in Japan, a society that values traditional educational credentials, graduating from a famous school/university is considered very important [48]. Therefore, adolescents who prioritize this value might feel more congruent with the community.

Regardless of the values that people prioritize, commitment to the values they considered important was independently associated with better subjective well-being both in Japanese and US participants. The results suggest that people who commit themselves to their own intrinsic values could benefit in adulthood, even if those values are generally extrinsic or not prevalent in their community. Because we did not investigate the interactions between value priorities and degrees of commitment to those values, further studies are needed to elucidate these interrelationships.

This study has several limitations. First, since we investigated the associations using a cross-sectional design, a causal relationship could not be determined. Second, all measures were self-reported, and therefore, may entail information bias and measurement errors. Personal values in adolescence were reported from remembrances, which could have been distorted by recall bias. Furthermore, the 11-item scale for personal values was developed in house and not completely validated. Although the other published study used the same scale, certain information bias might be caused by this measurement. Third, we could not calculate the valid response rate. This limitation might have led to a selection bias. For instance, unhealthy participants might have been reluctant to participate in the survey. If they had priorities for specific values, the positive associations between these values and mental health and well-being could have been overestimated. Fourth, the results between the countries could not be directly compared because of significant demographic differences. US participants were more likely than Japanese participants to be highly educated, regular/full-time managerial/professional workers than Japanese participants. These demographic differences might cause a difference in the results. Participants were not a random sample of workers, but were respondents to an Internet survey company. Therefore, we cannot generalize our findings to all Japanese and American workers. Finally, we limited the concept of “adolescence” to 15 to 16 years of age. Generally, “adolescence” describes children aged 10 to 25 [49]. We asked the participants to recall when they were 15 to 16 years old because that is the core age of the definition of adolescence [49].

## Conclusions

Certain personal values and commitment to those values in adolescence may be associated with mental health and well-being in the future. Care, graduating from school, and commitment to one’s values are suggested to be associated with better mental health and well-being both in Japan and the US. Belief and challenging may be associated with better mental health and well-being only in the US. On the other hand, financial success may be associated with poor mental health and well-being only in Japan. Avoiding causing trouble and positive evaluation may be associated with poor mental health and well-being only in the US. Further studies are needed to address the limitations of this study through use of a longitudinal design and investigation of the interactions among different types of personal values, and commitment to those values.

## Data Availability

The datasets used and analyzed during the current study are available from the corresponding author on request.

## Abbreviations

UN: United Nations
SDGs: Sustainable Development Goals
HRQoL: Health-related quality of life
PVQ-57: The 57-item Portrait Values Questionnaire
PBVS-C: The Picture-Based Value Survey for Children
PVQ-II: The Personal Values Questionnaire II
SF-8: The 8-item Short-Form Health Survey
SES: Socioeconomic status
